# xCT inhibition sensitizes tumors to γ-radiation via glutathione reduction

**DOI:** 10.18632/oncotarget.25794

**Published:** 2018-08-17

**Authors:** Lara Cobler, Hui Zhang, Poojan Suri, Catherine Park, Luika A. Timmerman

**Affiliations:** ^1^ Helen Diller Family Comprehensive Cancer Center, University of California, San Francisco, CA, USA; ^2^ Department of Radiation Oncology, University of California, San Francisco, CA, USA; ^3^ University of California, San Francisco, CA, USA

**Keywords:** radiation sensitize, glutathione, xCT, SLC7A11, radiation therapy

## Abstract

About 3 million US cancer patients and 1.7 million EU cancer patients received multiple doses of radiation therapy (RT) in 2012, with treatment duration limited by normal adjacent tissue damage. Tumor-specific sensitization could allow treatment with lower radiation doses, reducing normal tissue damage. This is a longstanding, largely unrealized therapeutic goal. The cystine:glutamate exchanger xCT is expressed on poor prognosis subsets of most solid tumors, but not on most normal cells. xCT provides cells with environmental cystine for enhanced glutathione synthesis. Glutathione is used to control reactive oxygen species (ROS), which are therapeutic effectors of RT. We tested whether xCT inhibition would sensitize xCT^+^ tumor cells to ionizing radiation. We found that pretreatment with the xCT inhibitor erastin potently sensitized xCT^+^ but not xCT^−^ cells, *in vitro* and in xenograft. Similarly, targeted gene inactivation also sensitized cells, and both modes of sensitization were overcome by glutathione supplementation. Sensitization prolongs DNA damage signaling, increases genome instability, and enhances cell death, revealing an unforeseen role for cysteine in genome integrity maintenance. We conclude that an xCT-specific therapeutic would provide tumor-specific sensitization to RT, allowing treatment with lower radiation doses, and producing far fewer side effects than other proposed sensitizers. Our data speaks to the need for the rapid development of such a drug.

## INTRODUCTION

Nearly 2/3 of all cancer patients in the United States receive radiation therapy (RT), comprising at least 3 million patients per year [1], including about ½ of breast cancer patients [2]. In the EU, an estimated 1.7 million cancer patients had an indication for radiation therapy in 2012, and that number is projected to grow to about 2 million in patients by 2025 [3]. While instrumentation now more specifically targets tumors, RT effects on normal tissues remain dose limiting [4], and treatment morbidity remains problematical [5–8]. Thus the need for therapeutics to specifically sensitize tumors and spare surrounding normal tissues is acute, and could benefit a huge patient population.

Ionizing radiation (IR) provides therapeutic benefit by terminally damaging DNA, in part by the generation of reactive oxygen species (ROS; [9]). However, the cellular DNA damage response proteins (DDR; review: [10]) sense DNA damage, halt proliferation, assemble repair complexes, and attempt DNA repair to maintain viability. Double strand breaks (DSBs) are particularly deleterious and are rapidly trimmed and ligated to any other available DNA end via non-homologous end joining (NHEJ). This can produce mutations at the repair joint and potential ligation of chromosome fragments to new partners, creating di-centric chromosomes and chromosomes of abnormal DNA content. Fragments that fail this capture (micronuclei) assort randomly during mitosis, increasing aneuploidy, and micronuclei frequency is used as a common measure of DNA damage and chromosome instability. Unresolved DNA integrity problems are propagated with subsequent mitoses, for example di-centric chromosomes may be mis-segregated, re-broken as daughter cells struggle over chromosome possession, or cause cytokinesis abortion to produces cells of high and abnormal ploidy (review: [11]). Cells with terminally damaged DNA succumb to fates including mitotic catastrophe and various types of programmed cell death or senescence (apoptosis, necrosis, necroptosis; reviews: [12, 13]). However, cells damaged in late S-phase or G2, where chromosomes are aligned, can repair DNA error-free by homologous recombination (HR) mitigating the therapeutic effects of RT.

Chromatin modification is one of the earliest steps in DNA repair, allowing focal recruitment of repair complexes to sites of damage. Key modifications include histone H2A phosphorylation (S-139; termed γ-H2AX; [14]), and loading of tumor protein 53 binding protein 1 (53BP1) dimers onto freshly ubiquitinated histone H2A and constitutively mono and di-methylated histone H4 (review: [15]). 53BP1 competes with breast cancer associated 1 (BRCA1) to bind chromatin and promote NHEJ by preventing the extensive DNA end re-sectioning that is required for HR-mediated repair. Upon repair completion, H2AX is dephosphorylated and foci are disassembled (review: [16]). Disassembly of 53BP1 foci is less well understood, but involves ubiquitin ligases and acetylation or phosphorylation at adjacent chromatin sites (review: [15]).

Intracellular antioxidants rapidly neutralize ROS to minimize DNA damage and the need for DNA repair, primarily by oxidation of intracellular thiol-containing substrates (review: [17]). Glutathione comprises roughly 90% of intracellular non-protein thiols, and is maintained at millimolar concentrations in a reduced form (GSH) in cells (review: [18]). GSH synthesis is limited by intracellular cysteine abundance, which is derived from the diet or synthesized from methionine transsulfuration. GSH is oxidized to a disulfide-linked homodimer (GSSG) by glutathione peroxidases during ROS inactivation. Other antioxidants such as the thioredoxin and peroxyredoxin reductases also rely on access to abundant thiols, via the amino acid cysteine and glutathione. Although radiation protection by cysteine/thiol supplementation has been recognized since biological studies of radiation exposure were initiated [19, 20], the development of protectants for normal cells and/or specific tumor sensitizers has been stymied by the inability to separately effect tumor versus surrounding normal tissues (review: [4]).

Antioxidant stress can cause the demand for glutathione and thiol synthesis to outstrip endogenous cysteine supplies [21, 22]. In the naturally oxidizing extracellular environment, cysteine is predominantly found as a disulfide-linked homodimer (cystine). Except in the brain, the cystine:glutamate exchanger xCT (encoded by *SLC7A11)* is the sole transporter that allows access to this amino acid reservoir [22]. xCT is transcriptionally induced via stress response signaling factors KEAP1/NRF2 [23] in response to glutathione demands [21]. Pathway activating mutations are found in breast [24], lung [25, 26], esophageal [27], and biliary tract [28] tumors, and confer radiation resistance [29]. xCT is also induced in response to insulin-like growth factor 1 signaling in estrogen receptor positive breast cancer cells [30], and during amino acid starvation response to activation of the transcription factor ATF4 [31, 32].

xCT is expressed by few normal human tissues except brain [33, 34], and is dispensable for fetal development, and adult viability and fertility [35–37]. In contrast, subsets of most solid tumors express xCT, and expression independently predicts poor clinical responses in glioma [38], glioblastoma [39, 40], esophageal [41], hepatocellular [42, 43], colorectal [44], prostate [45] lung [46] and breast [30] carcinomas. We previously found that about ½ of triple negative breast cancer (TNBC) clinical specimens and TNBC-derived cell lines overexpress xCT/*SLC7A11* [47]. We demonstrated that xCT inhibition via off-target activity of the bowel anti-inflammatory sulfasalazine (SASP) reduced GSH levels, increased endogenous ROS, and strongly reduced growth of xCT^+^ triple negative breast cancer (TNBC) lines *in vitro* and in xenograft. Here we test the hypothesis that targeting the *SLC7A11* gene, or treatment with the xCT inhibitor erastin, will reduce intracellular thiols and produce specific IR sensitization of xCT^+^ but not xCT^−^ cells.

## RESULTS

### *SLC7A11* gene targeting prevents clonogenic colony formation and tumor formation in xenograft

**Figure 1:**
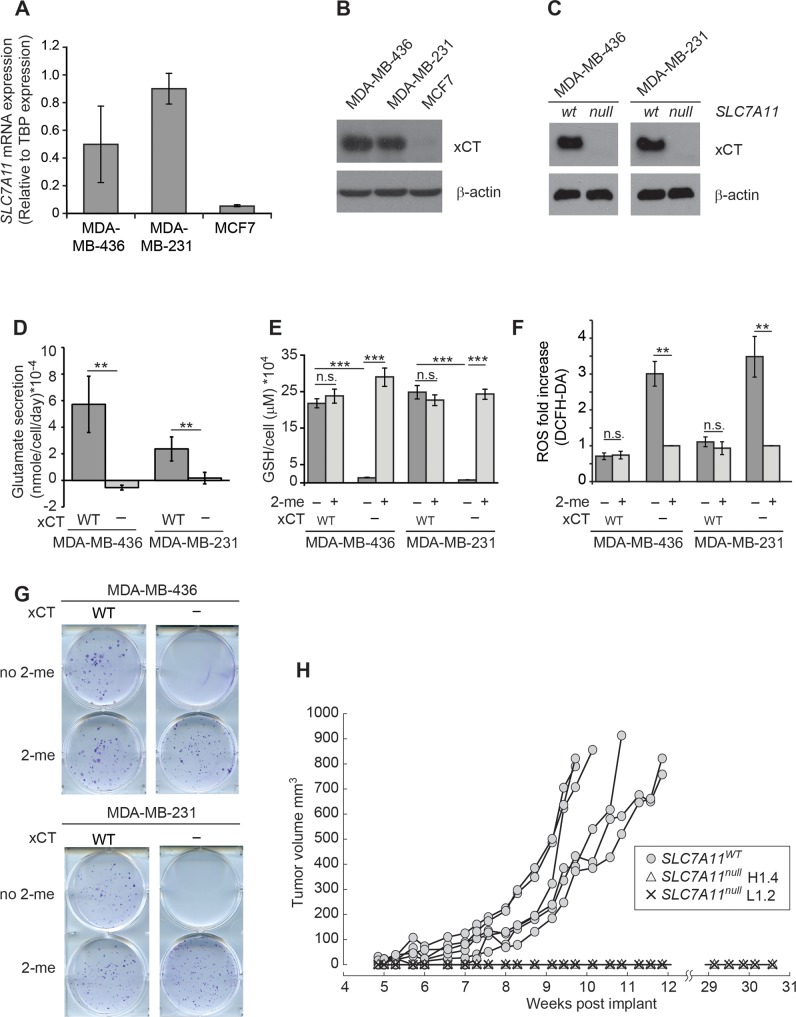
*SLC7A11* targeting reduces intracellular glutathione and prevents growth *in vitro* and in xenograft **(A)** *SLC7A11* mRNA levels assessed by quantitative RT-PCR. **(B)** Expression of the protein product of *SLC7A11* (xCT) assessed by western blot. **(C)** Comparison of xCT protein levels in cells with intact *SLC7A11* versus pooled subclones with *SLC7A11*-targeted mutation. **(D-H)** Analysis of cell lines with intact *SLC7A11* (WT) versus pooled *SLC7A11*-targeted subclones (−). (D) Glutamate levels in culture media after 24 hours. (E) Total intracellular glutathione in cells cultured 24 hours cultured without 2-me versus with 2-me to allow cysteine import as mixed dimers via transporters other than xCT. (F) ROS levels assessed by DCFH-DA staining (10μM) and FACS analysis in cells cultured for 24 hours without 2-me, normalized to culture with 2-me. (G) Colony formation ability of cells cultured with and without 2-me. Experiments used 3-6 replicates, performed 2-4 times. (H) Growth curves of MDA-MB-231 cells in xenograft contrasting intact *SLC7A11* (WT), versus two independent MDA-MB-231 subclones with *SLC7A11*-targeted mutation (H1.4 and L1.2), 6 mice/group. All values are means +/− SD. *t*-test significance; n.s. not significant; ^*^, p<=0.05; ^**^, p<=0.01; ^***^, p<=0.001.

Breast cancer cell lines were selected based on expression of the gene encoding xCT (*SLC7A11*) using RT-qPCR (Figure 1A), and xCT protein levels by western blot (Figure 1B). MCF7 expresses little if any xCT, and is used as a negative control in these studies. MDA-MB-231 (M231) is homologous recombination repair (HR) competent, while MDA-MB-436 (M436) is incompetent due to homozygous BRCA1 mutation [48, 49]. xCT negative variants of M231 and M436 were derived by targeting *SLC7A11* (*SLC7A11*^null^), which prevented protein expression (Figure 1C). These *SLC7A11*^null^ variants are maintained in media containing 2-mercaptoethanol (2-me), which allows cysteine import as mixed disulfides using transporters other than xCT [50]. Culture in 2-me-free media forces these variants to become reliant on xCT-mediated cystine:glutamate exchange for cystine acquisition, and produces significantly reduced glutamate secretion and intracellular glutathione levels (Figure 1D–1E), with correspondingly higher levels of endogenous reactive oxygen species (ROS; Figure 1F). Unlike the acute ferroptotic cell death observed by others in some cell lines [51], we observed little/no cell death 24 hours after 2-me withdrawal (Supplementary Figure 1), however the *SLC7A11*^null^ variants of both cell lines are unable to form colonies in standard clonogenic assays used to test radiation sensitizers (Figure 1G). Similarly, M231 *SLC7A11*^null^ variants do not grow in xenograft (Figure 1H).

### Erastin inhibits xCT activity in xCT positive breast cancer cell lines

**Figure 2:**
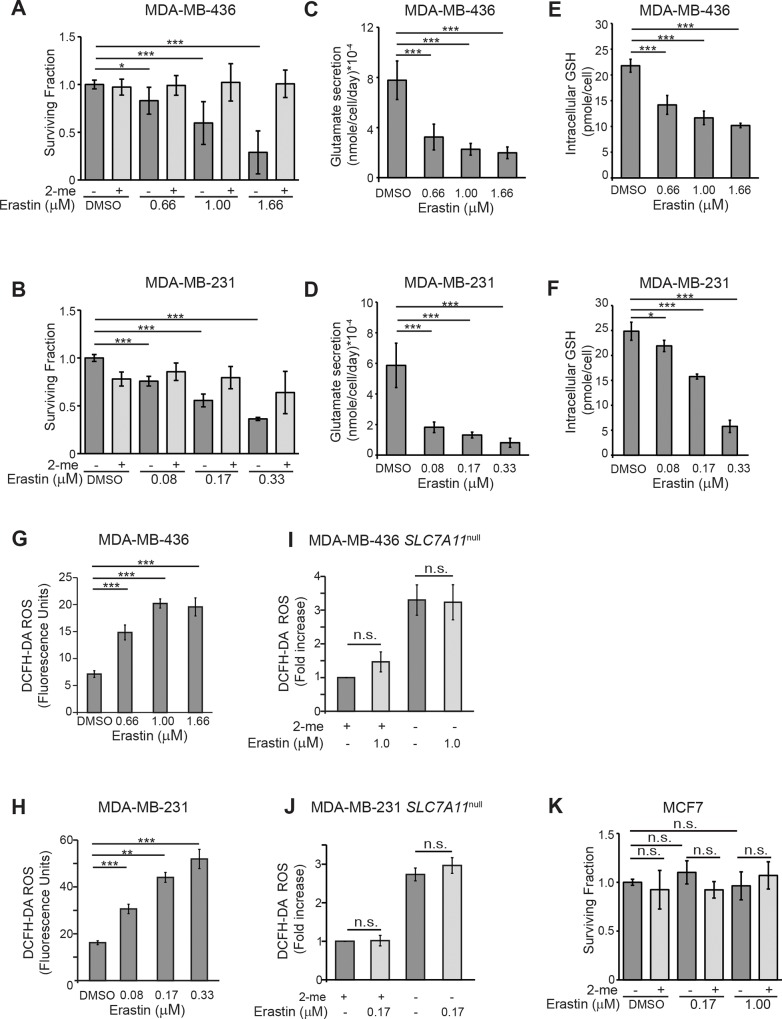
Erastin inhibits xCT activity in xCT+ breast cancer cell lines **(A, B)** Erastin concentrations (IC) for growth inhibition in clonogenic assays (dark grey bars). Values are: MDA-MB-436 IC_25_, 0.66μM; IC_50_,1.0μM; IC_75_,1.66μM. MDA-MB-231 IC_25_, 0.08μM; IC_50_, 0.17μM; IC_75_, 0.33μM. Erastin treatment with 2-me addition (light grey bars) to allow cysteine uptake without xCT use largely prevents erastin growth inhibitory effects. **(C-J)** Phenotypic changes associated with xCT inhibition by 24 hours of erastin treatment. (C, D), Glutamate abundance in culture media. (E, F), Total intracellular glutathione. (G, H), Intracellular ROS levels assessed by DCFH-DA staining (10μM) and FACS analysis. (I, J), Erastin treatment (IC_50_) of pooled *SLC7A11*^null^ clones and analysis of ROS levels in cultures with (light grey bars) or without (dark grey bars) 2-me supplementation. Values normalized to 2-me samples. **(K)** Erastin treatment of cells that are naturally xCT negative (MCF7) does not produce growth defects in clonogenic assays at the IC_50_ for MDA-MB-231 (0.17μM), or MDA-MB-436 (1.0μM), without (dark grey bars) or with (light grey bars) 2-me supplementation. Experiments used 3-6 replicates, performed 2-5 times. Figures are means +/− SD. *t*-test significance; n.s., not significant; ^*^, p<=0.05; ^**^, p<=0.01; ^***^, p<=0.001.

Since the *SLC7A11*^null^ variants do not grow in xenograft and cannot form colonies in clonogenic assays, (Figure 1G–1H) we used the xCT inhibitor erastin to test whether an xCT-targeted therapeutic could be used to sensitize tumor cells to ionizing radiation (IR). Dose response curves were generated for colony formation in the xCT^+^ lines M436 and M231 and the concentrations that inhibit colony by 25% (IC_25_), 50% (IC_50_), and 75% (IC_75_) were calculated (Figure 2A, 2B dark bars). These colony formation defects were largely corrected if culture media contained 2-me, indicating that the dominant anti-proliferative activity of erastin in these cells at these doses was restriction of extracellular cystine access (light bars). Accordingly, erastin treatment also reduced glutamate secretion, (Figure 2C, 2D), decreased intracellular GSH concentrations (Figure 2E, 2F) and increased intracellular ROS (Figure 2G–2H) in a dose-dependent fashion. These data indicate that xCT is a major intracellular target of erastin, as previously described in other tumor cell types [52]. Specificity for xCT was further tested by treating our *SLC7A11*^null^ variants with erastin at the clonogenic IC_50_ of each parental cell line (M436, 1μM; M231, 0.17μM; Figure 2I-2J). This produced no further significant increase in ROS in the presence or absence of 2-me. Similarly, treatment of a naturally xCT negative cell line (MCF7) at the M436 or M231 clonogenic IC_50_ concentrations produced no colony formation defects (Figure 2K) and little increase in ROS (Supplementary Figure 2). We conclude that at these doses and in these xCT positive cell lines, our assays are reporting xCT inhibition by erastin.

### Erastin treatment sensitizes breast cancer cells to IR

**Figure 3:**
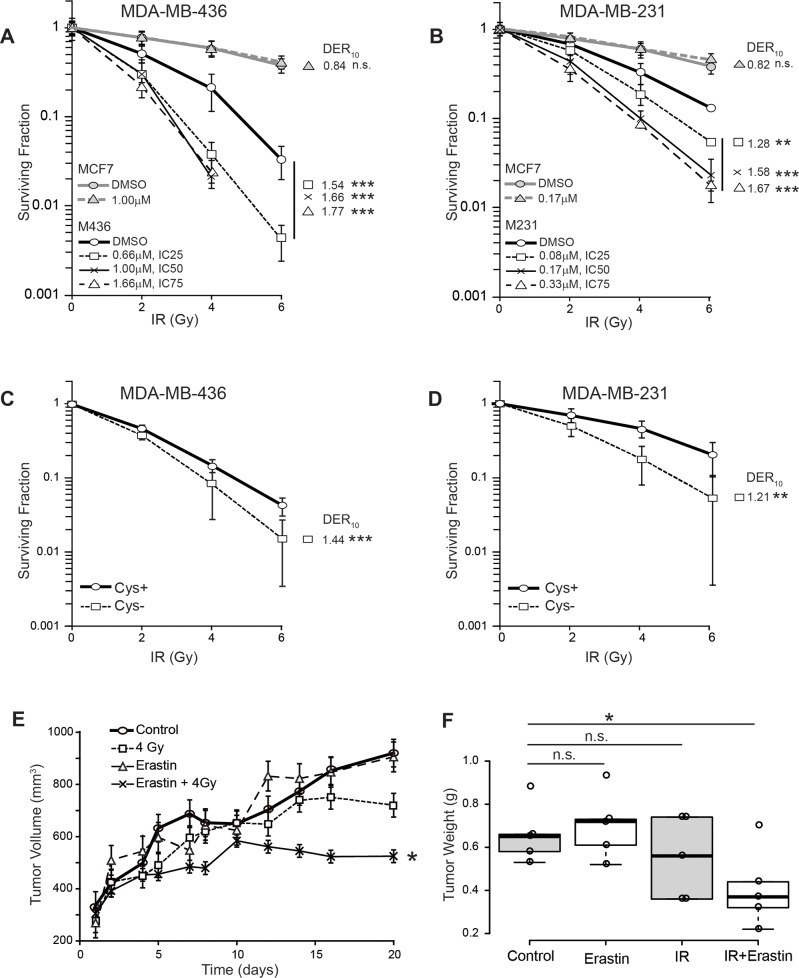
Erastin sensitizes xCT+ cells to ionizing radiation (IR) *in vitro* and in xenograft **(A-D)** Survival curves and radiation dose enhancement ratios (DER_10_). DER_10_ >1 indicates enhanced sensitivity. (A) Erastin pre-treatment of MDA-MB-436 (black), compared to MCF7 (grey). MDA-MB-436 IC_25_, 0.66μM; IC_50_,1.0μM; IC_75_,1.66μM. (B) Erastin pre-treatment of MDA-MB-231 (black), compared to MCF7 (grey), IC_25_, 0.08μM, IC_50_, 0.17μM, IC_75_, 0.33μM. (C, D) Survival curves for cells cultured in cystine replete, versus cystine-free media. Experiments performed at least twice in triplicate. **(E, F)** MDA-MB-436 xenografts given erastin (16.5 mg/kg) or vehicle control (DMSO/PBS) pre-treatment; 4 Gy partial body irradiation or sham. Erastin (16.5 mg/kg) continued daily. (E) tumor growth curves (mean +/− SEM). (F) Boxplot center lines are median tumor weights; box limits indicate the 25th and 75th percentiles (R software); whiskers extend 1.5 times the interquartile range from the 25th and 75th percentiles; data points are open circles. *t*-test significance; n.s. not significant; ^*^, p<=0.05; ^**^, p<=0.01; ^***^, p<=0.001.

Clonogenic survival curves for xCT^+^ and xCT^−^ breast cancer cell lines were generated, testing the ability of 16 hour erastin pretreatment to sensitize tumors to γ-radiation doses spanning 0-6 Gray (Gy). This pretreatment produced no increase cell death (Supplementary Figure 3A, 3B) or changes in cycle profiles at 16 hours (Supplementary Figure 3C, 3D). Calculation of the surviving fraction at each erastin/radiation dose combination and the dose enhancement ratios at 10% survival (DER_10_) revealed that erastin pretreatment significantly sensitized the xCT^+^ cell lines M436 and M231 to IR (Figure 3A, 3B black lines). For example, at the erastin IC_50_s, the IR dose required to achieve DER_10_ is reduced by about 66% in M436 and 58% in M231 (M436 DER_10_=1.66; M231 DER_10_=1.58). Conversely, MCF7 (xCT-) exhibited little if any sensitization by pre-treatment at these doses (Figure 3A, 3B, grey lines; DER_10_ at 1uM=0.84; and at 0.17uM=0.82 respectively). We verified that sensitization was due to cystine limitation by using cystine-free media rather than erastin treatment, which also sensitized cells (Figure 3C, 3D; M436 DER_10_=1.44, M231 DER_10_ =1.21). Finally, we tested erastin sensitization of M436 in orthotopic xenografts (Figure 3E, 3F). Erastin + IR produced significantly smaller tumors than the control group (p=0.04), while IR alone (p=0.333) or erastin treatment alone (p=0.658) did not. Thus xCT inhibition induces significant IR sensitization *in vitro* and *in vivo*.

### Erastin sensitization prolongs radiation-induced DNA damage signaling

**Figure 4:**
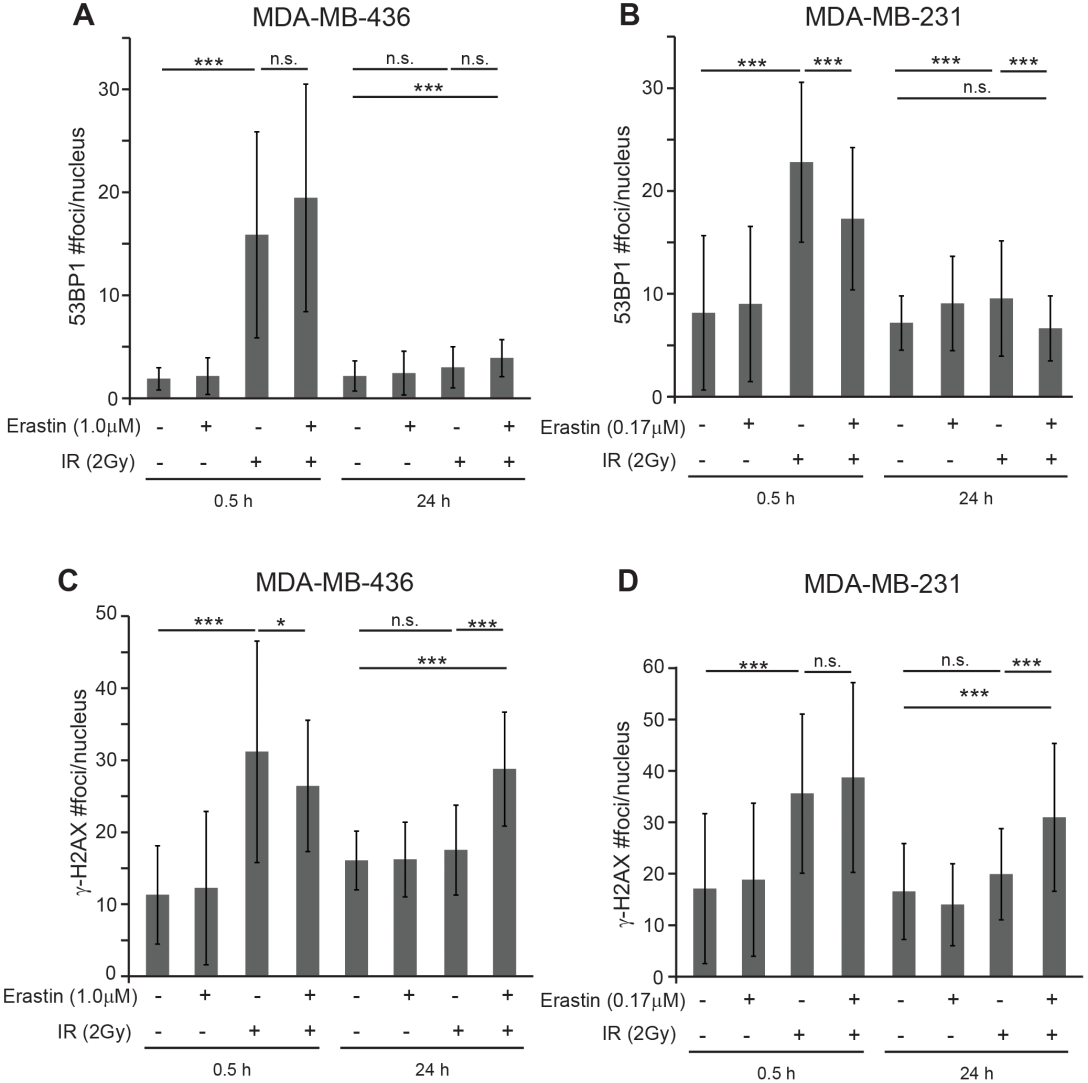
Erastin sensitization produces sustained γ-H2AX foci Nuclear foci counts in MDA-MB-231 and MDA-MB-436 cells given 16 hours erastin at the clonogenic IC_50_ or DMSO pretreatment before 2 Gy IR. MDA-MB-436 IC_50_, 1.0μM; MDA-MB-231 IC_50_, 0.17μM. **(A, B)** 53BP1 foci/nucleus 0.5 and 24 hours after IR. **(C, D)** g-H2AX foci/nucleus 0.5 and 24 hours after IR. Foci assessed by immune fluorescence and microscopic quantitation. At least 50 nuclei per condition evaluated. Experiments performed in triplicate three times. Figures are means +/− SD. *t*-test significance; n.s., not significant; ^*^, p<=0.05; ^**^, p<=0.01; ^***^, p<=0.001.

Since our data revealed that xCT inhibition by both chemical and genetic means reduces glutathione levels and increases intracellular ROS (Figure 1E, F; Figure 2E-2J), we hypothesized that xCT inhibition might sensitize to IR by enhancing DNA damage. In timecourse studies using 2 Gy and erastin at the IC_50_ doses, we determined the abundance and duration of DNA repair foci containing the chromatin remodeling complexes 53BP1 and γ-H2AX. We found that 30 minutes post IR, 53BP1 foci numbers are increased in irradiated samples of both lines as expected (Figure 4A–4B, 0.5h). In M436 there was no significant difference between sensitized + irradiation versus irradiation alone, while in M231 the differences were significant but represent only a slight focus formation delay in the erastin + IR samples versus the IR alone samples (Supplementary Figure 4; complete time course example). At 24 hours post IR, 53BP1 foci are reduced essentially to control levels In both cell lines suggesting that the majority of double strand breaks are repaired, although possibly incorrectly (Figure 4A, 4B, 24 h). Thus erastin does not sensitize cells by preventing formation or disassembly of 53BP1 foci.

**Figure 5:**
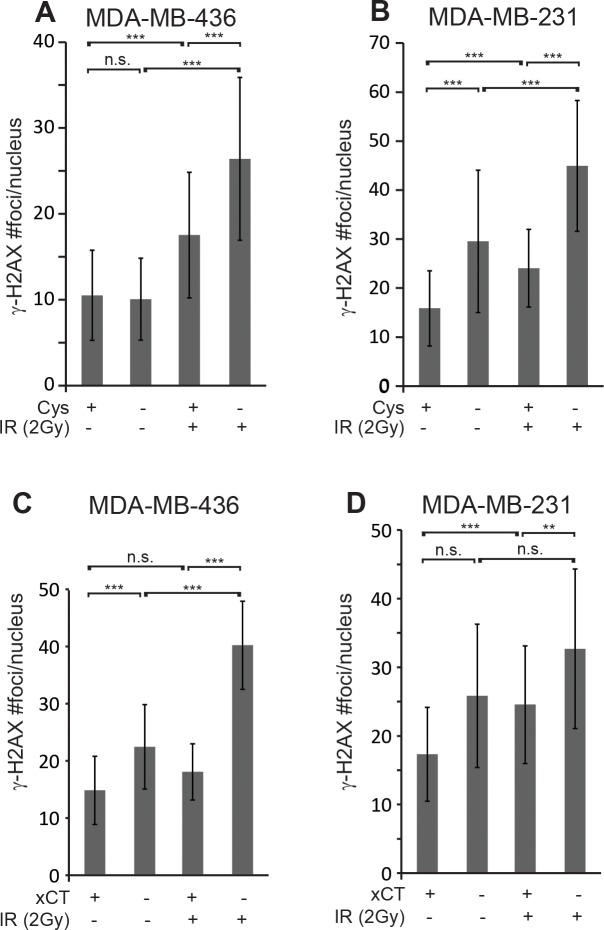
Cystine starvation and *SLC7A11*-targeted inactivation produce sustained γ-H2AX foci **(A, B)** γ-H2AX foci/ nucleus 24 hours post 2 Gy IR in MDA-MB-436 and MDA-MB-231 cells that were cystine starved 16 hours before IR. **(C, D)** γ-H2AX foci/ nucleus in *SLC7A11*^wt^ (xCT+) versus *SLC7A11*^null^ (xCT-) cells cultured without 2-me for 16 hours before 2 Gy IR. Foci assessed by immune fluorescence and microscopic quantitation. At least 50 nuclei per condition evaluated. Experiments performed in triplicate three times. Figures are means +/− SD. *t*-test significance; n.s., not significant; ^*^, p<=0.05; ^**^, p<=0.01; ^***^, p<=0.001.

Analysis of γ-H2AX staining also revealed little if any difference in foci numbers between the irradiated versus erastin sensitized, irradiated samples at 30 minutes (Figure 4C–4DC, 0.5h; M436 p=0.05; M231 p= n.s.). However 24 hours post 2 Gy IR we found significantly more unresolved foci in the erastin sensitized, irradiated samples versus the irradiation alone samples, indicative of unresolved DNA damage (24h; M436 p=0.0001; M231 p=0.002). This is not due to differences in cell death (Supplementary Figure 5A, 5B) or cell cycle distribution (Supplementary Figure 5C, 5D). Thus erastin sensitization changes the duration of some IR-induced DNA damage complexes, suggesting that it may produce more profound DNA damage.

### Cystine starvation and *SLC7A11*-targeted inactivation also prolong the duration of γ-H2AX foci

To verify that the persistent γ-H2AX foci in the erastin sensitized samples are due to a cystine deficit, we tested whether culture in cystine deficient media would prevent timely resolution of γ-H2AX foci. In agreement with the erastin data, cystine restriction left significantly more IR-induced γ-H2AX foci at 24 hours post IR than cystine replete media in both cell lines (Figure 5A, 5B; M436 p=0.00001; M231 p=0.00001). Cystine starvation alone also produced significantly more foci in M231 cells than culture in cystine replete media (p=0.0008), while this was not seen in M436.

To provide direct genetic evidence for the role of xCT inhibition in prolonging IR-induced γ-H2AX foci, we counted foci numbers in irradiated *SLC7A11*^wt^ versus *SLC7A11*^null^ clones of M436 and M231. Analysis of γ-H2AX foci revealed clearly and significantly elevated foci numbers 24 hours post IR in both *SLC7A11*^null^ cell lines versus their *SLC7A11*^wt^ counterparts, similar to erastin pretreatment (Figure 5C, 5D; M436 p=0.00001; M231 p=0.009). The *SLC7A11*^null^ variants of both lines also exhibit increased numbers of γ-H2AX foci without irradiation versus their *SLC7A11*^wt^ counterparts, suggesting that xCT loss may impart some challenge to maintenance of genome integrity. This data indicates xCT inhibition by genetic or chemical means alters the quality of IR-induced DNA damage responses.

### Radiation sensitization induces genome instability

**Figure 6:**
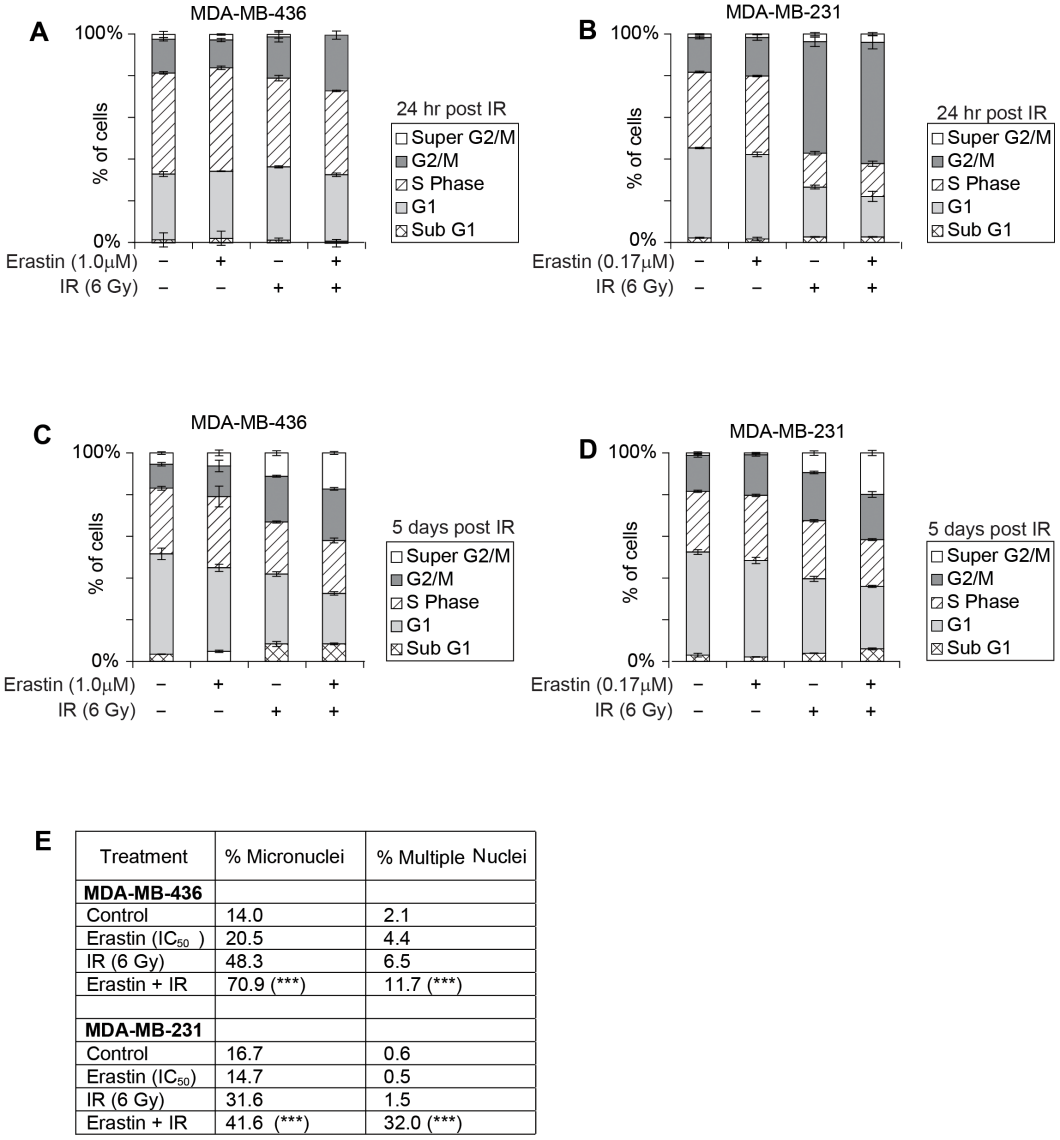
Erastin sensitization and *SLC7A11*-directed mutation increase radiation-induced cell death and genome instability Erastin 16 hour pre-treatment at the clonogenic IC_50_: MDA-MB-436, 1.0μM; MDA-MB-231, 0.17μM; IR, 6 Gy. **(A-D)** Cell cycle profiles of indicated treatment groups assessed by PI staining and FACS analysis, performed 3-5 times with 50,000 - 100,000 cells analyzed per condition. (A, B), 24 hours post IR. (C, D), 5 days post IR. **(E)** Quantitation of day 5 nuclear morphologies, assessed by DAPI stain and microscopic evaluation of 1000 nuclei per sample. _X_^2^ significance; ^***^, p<=0.001 for IR alone versus Erastin sensitization +IR.

If DNA damage was potentiated by erastin pre-treatment before IR, then more chromosomal abnormalities and cell death should become apparent as cells repeatedly transit the cell cycle. We examined these features in our treatment groups over a 5 day timecourse. Similar to most cancer cells, neither M436 nor M231 exhibit a strong G1 arrest 18-24 hours post IR (Figure 6A, 6B). M436 exhibits a marginally increased G2/M fraction (Figure 6A, maximum 6% increase), while IR induces a strong G2/M arrest in M231 with or without erastin sensitization (Figure 6B). Thus erastin does not prevent G2 DNA damage checkpoint activation.

Two days post IR, the M231 cell cycle profiles normalized (Supplementary Figure 6A 5-7% maximum differences), and the G2/M fraction of M436 sensitized, irradiated cells expanded incrementally relative to other treatment groups, with corresponding reduction in the G1 fraction (Supplementary Figure 6B, G2/M 30% versus 22-17% in other treatment groups, G1 29% versus 40-44% in other treatment groups). These features suggested ongoing proliferation, which was verified by the appearance of a prominent nocodazole-induced G2/M fraction in each treatment group of both cell lines on day 3 (Supplementary Figure 6C, 6D). Thus erastin sensitization does not prevent cells from progressing through several rounds of mitosis after IR.

At day 5 post IR, cell cycle profiles revealed more severe G1 fraction loss in the sensitized, irradiated samples (M436 G1 size: 24.2% versus 33-47% in other treatment groups; M231 30% versus 36-50%), and an increase in the > 4N DNA fraction with erastin sensitization (Figure 6C, 6D, “Super G2M”; M436, 17% versus 11.2-5.4%; M231, 20% versus 9.4-0.9% in other treatment groups). The appearance of cells with greater than 4N DNA content is a hallmark of genomic instability, as is the increase in the appearance of chromatin bridges, micronuclei, and multinucleated cells. We quantitated the frequency of the latter two morphologies and found that they were significantly more prevalent in the sensitized, irradiated samples (Figure 6E). Thus, erastin sensitization produces increases genome instability upon IR exposure yielding highly abnormal nuclear content after several rounds of mitosis, in accord with a role in potentiating radiation-induced DNA damage effects.

### RT sensitization by xCT inhibition increases glutathione sensitive cell death

**Figure 7:**
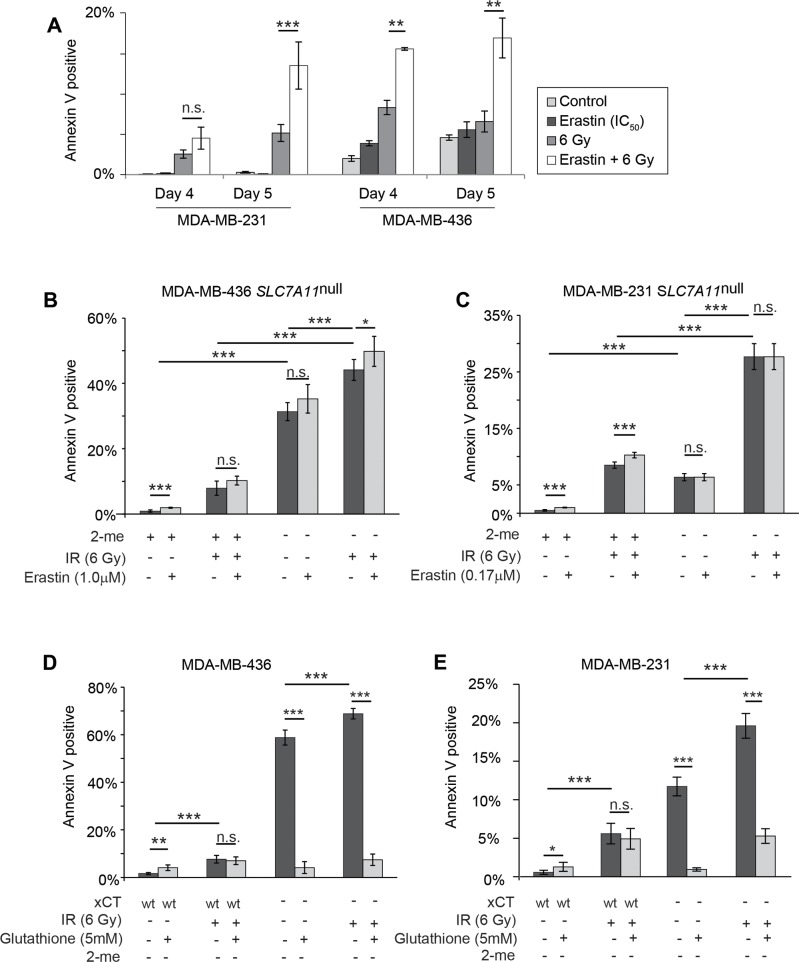
Erastin sensitization and *SLC7A11*-targeted mutation increase radiation-induced cell death in a glutathione-sensitive fashion Percent Annexin V positive cells determined by Annexin V /PI staining and FACS analysis of 10,000-50,000 cells per condition, 4 and 5 days post IR treatment. **(A)** 16 hour erastin pre-treatment at the clonogenic IC_50_ (MDA-MB-436, 1.0μM; MDA-MB-231, 0.17μM) increases annexin V staining at day 5 post IR. **(B, C)** Culture without 2-me increases annexin V staining of *SLC7A11*^null^ subclones at day 5 post IR. Erastin treatment does not further increase the annexin V positive fraction. **(D, E)** Glutathione (5mM) effects on Annexin V levels at day 5 post IR, in *SLC7A11*-intact (WT) versus pooled *SLC7A11*^null^ (−) subclones. Experiments used 3-6 replicates, performed 2-4 times. *t*-test significance; n.s. not significant; ^*^, p<=0.05; ^**^, p<=0.01; ^***^, p<=0.001.

Over days 1-3 post IR, we observed only minor increases in annexin V staining in all treatment groups in both MDA-MB-436 and MDA-MB-231 (<4%; Supplementary Figure 6E-6F, maximum 7%). However, significant apoptosis (Annexin V^+^/PI^−^ staining) became apparent at days 4 and 5, and was significantly higher in erastin sensitized, IR treated samples (Figure 7A). Genetic evidence for xCT involvement was demonstrated by withdrawal of 2-me from M436 *SLC7A11*^null^ cultures with and without subsequent IR treatment. Four days of culture without 2-me produces significant increases in Annexin V reactive cells in (Figure 7B). However 2-me withdrawal for 16 hours followed by IR treatment still produces significantly more death than 2-me withdrawal alone (M436 31.3% versus 44.1%) or IR treatment in the presence of 2-me (7.9% versus 44.1%). M231 *SLC7A11*^null^ cultures are similarly sensitized (Figure 7C). We confirmed that erastin-mediated IR sensitization in *SLC7A11^wt^* cells was largely due to on-target effects by adding erastin (IC_50_) to the treatment conditions of the *SLC7A11*^null^ variants. This produced either insignificant or significant but small (5% maximum) increases in cell death (Figure 7B, 7C; dark versus light grey bars). We conclude that 2-me withdrawal in the *SLC7A11*^null^ cells and erastin pretreatment in *SLC7A11*^wt^ cells both primarily limit extracellular cystine access by preventing xCT function, sensitizing cells to subsequent IR.

Finally, we tested whether glutathione supplementation would alter sensitization to IR-induced cell death, since glutathione is intimately involved in ROS control, and intracellular levels are reduced by erastin treatment or xCT knockdown (Figure 1E; Figure 2E, 2F). We found that glutathione supplementation did not significantly alter the percent of Annexin V positive cells in irradiated *SLC7A11*^wt^ cultures at day 5, suggesting that these cells already make sufficient glutathione to maximize their metabolic ability to control IR-induced death (Figure 7D, 7E). However analysis of *SLC7A11*^null^ cells cultured without 2-me revealed that glutathione supplementation significantly reduced death, both in cultures simply lacking 2-me, and in 2-me-starved cultures treated with IR. We conclude that development of a clinically-approved xCT inhibitor could be used to potentiate RT-induced DNA damage and tumor killing. Since the most normal cells do not express xCT, a specific xCT inhibitor would provide a means to specifically sensitize tumors to RT.

## DISCUSSION

### Most radiation protectants and sensitizers lack tumor specificity

Observations that acute pre-treatment cysteine injection can protect rats from radiation-induced death are now more than 60 years old [19], and attempts to develop clinically-applicable radiation protectants for normal tissues continues to date. Enhancement of normal tissue GSH levels initially received the most therapeutic effort [53, 54], although the highly elevated levels of glutathione in tumors challenge this strategy. Amfostine is currently the only radiation protectant in clinical use. This prodrug is activated by high level of alkaline phosphatases in normal cells, versus tumors (reviewed in: [55]). Other potential antioxidant-based protectants include nitroxides such as Tempol, alpha tocopherol, or beta carotene. Melatonin can enhance production of endogenous antioxidants, and antibiotics such as tetracycline and ciprofloxacin influence chromatin remodeling and enhance DNA repair [56]. However, most of these compounds show little therapeutic window for normal tissues over tumors [55, 57].

Efforts to develop tumor-specific sensitizers are conversely stymied by an inability to sensitize tumors without sensitizing normal tissues. Early work targeted the glutathione synthetic enzyme gamma-glutamylcysteine synthetase (GGS; review: [58]) which unfortunately is ubiquitously and highly expressed. Other potential strategies include the use chemotherapy such as DNA damaging agents (example: 5-FU, cisplatin); compounds that slow/inhibit DNA repair (Fludarabine, doxorubicin); or agents that arrest tumor cells in the most radiation-sensitive portion of the cell cycle (G2; taxanes; review: [59, 60]). Potential molecular targets include DNA repair enzymes such as ATM, cell cycle checkpoint and survival signaling molecules such as CHK1, and EGFR/AKT/PI3K (review: [4]). Radiation induces local activation of TGFβ and β1 integrins, and inhibition of these molecules can potently sensitize in preclinical cancer models [61, 62]. Hypoxia prevents the therapeutic benefit of IR. Early hypoxic tumor sensitization strategies unsuccessfully tested glutathione depletion via nonspecific compounds (reviews: [63, 64]). Currently, hypoxia-activated prodrugs such as nitroimidazole are under development (review: [4]). However, the majority of these potential sensitizing targets are widely expressed, and the ultimate therapeutic window these strategies will create is unclear. In contrast, with the exception of brain, xCT is expressed in only a few normal tissues and at low levels (examples: thyroid, esophagus, stomach, fallopian tubes [34]). Importantly, three distinct strains of mice bearing an inactivated *SLC7A11* genetic locus have normal development, lifespan, and fertility- strongly suggesting that short term treatment with a clinically-approved xCT therapeutic should have minimal deleterious side effects (review: [65]).

### xCT inhibition provides a means to specifically sensitize xCT positive tumors to IR

We demonstrate that *SLC7A11*-targeted inactivation (Figure 7B–7E), pretreatment with the xCT inhibitory compound erastin (Figure 3A, 3B, 3E, 3F; Figure 7A), and culture in cystine-free media (Figure 3C–3D) provide potent radiation sensitization of xCT^+^ tumor cells, *in vitro* and in xenograft. Although other intracellular targets have been reported for erastin [66], in our cell lines and at the doses used in this study, we find that erastin effects are largely on-target: **a)** erastin-induced reduction in clonogenic colony formation is prevented by 2-me (Figure 2A, 2B); **b)** clonogenic colony formation of naturally xCT^−^ cells (MCF7) is not reduced with erastin treatment (Figure 2K); **c)** MCF7 are not sensitized to IR by erastin pretreatment (Figure 3A, 3B; grey lines, MCF7); and **d)** erastin addition to our *SLC7A11*^null^ cells cultured under restrictive or non-restrictive conditions (without or with 2-me) and with or without subsequent IR, does not significantly increase cell death (Figure 7B, 7C). Recently large doses of Sulfasalazine, a potent NFkB inhibitor and a weak competitive xCT inhibitor were used to sensitize gliomas to RT [67–69]. However effects on xCT^−^ cells were not tested, thus the key functional target in those studies remains unclear. In our M436 proof-of-concept xenografts, erastin sensitized to a single radiation dose (4 Gy), produced smaller tumors (Figure 3E, 3F). Typical conventional hypofractioned radiation regimens for human whole breast irradiation deliver 2 Gy fractions 5 times per week until 46-50 Gy have been delivered, or ∼2.7 Gy until 40-42 Gy is achieved [70]. Extension of the dose response IR curves (Figure 3A, 3B) to these higher total doses suggests that sensitization by xCT inhibition at each round of RT could have a profoundly beneficial effect on clinical radiation outcomes.

### xCT inhibition may result in more complex DNA damage

Thirty minutes after IR exposure, erastin sensitized samples have essentially the same numbers of γ-H2AX foci as the IR-alone samples (Figure 4C, 4D; 0.5h). This suggests that there are not significantly more visually dispersed loci damaged by erastin sensitization. However the prominent γ-H2AX foci remaining at 24 hours with erastin sensitization (Figure 4C, 4D; 24h) suggests that erastin prevented the resolution of many DNA abnormalities. These lingering foci can be directly attributed to xCT inhibition in that *SLC7A11*-targeting (Figure 5C, 5D) and cystine restriction (Figure 5A, 5B) also produce significantly more γ-H2AX foci 24 hours post IR.

Two possibilities may account for the lingering γ-H2AX foci in sensitized, irradiated cells. First, they may be due to unresolved SSBs. Both γ-H2AX and 53BP1 foci are produced during S-phase replication fork stalling and replication stress [71, 72], and persistent SSBs can be converted to DSBs during cell division, producing late-appearing, cumulative DNA damage and cell death. Alternatively, high linear energy transfer (LET) densely ionizing radiation such as γ-radiation can produce tracks of “regionally multiply damaged sites”, including multiple DSBs localized to short stretches of DNA ([73]; reviewed: [74]). Repair at these sites is difficult, and may provide the most therapeutic benefit. Non-proliferating mammary epithelial cells treated with high LET radiation maintain γ-H2AX foci up to 72 hours after treatment, while resolving 53BP1 foci by 48 hours [75]. Similar to our observation that persistent γ-H2AX foci, genome instability, and increased cell death occur in our sensitized cultures (Figure 4C, 4D; Figure 5A-5D), others have also correlated the number of cells with lingering γ-H2AX foci with the fraction of cells that ultimately die from unresolved DNA damage [76, 77]. This count may be a predictive biomarker of good radiation responses [78].

### RT treatment may particularly benefit tumors bearing somatic DDR gene mutations

Our xCT^+^ cell lines bear different mutations that impact DNA repair. M436 has inactivated p53, retinoblastoma, and BRCA1 ([48, 49]; cancer.sanger.ac.uk). BRCA1 loss compromises HR, increasing the potential for mutant chromosome generation by NHEJ-mediated repair. Unlike M231, M436 also do not enact a radiation-induced G2 arrest (Figure 6A versus 6B). Thus, although end ligation by NHEJ occurs rapidly, some M436 cells may undergo the first mitotic division after radiation with free chromosome fragments and improperly re-ligated fragments, increasing the frequency of DNA mis-segregation and generation of daughters with abnormal ploidy. In contrast, p53 is mutated but functional in M231, and BRCA1 is intact [48]; cancer.sanger.ac.uk). Transient arrest in G2 provides time for DNA damage correction by HR, and for capture of free chromosome fragments before mitosis. Accordingly, we find that M436 are more readily sensitized to RT than M231 (Figure 3A versus 3B, compare DER_10_ values). Sensitized and IR-treated M436 day 5 cultures also contain more cells with micronuclei and multiple nuclei (Figure 6E). We speculate that tumors bearing somatic mutation/silencing of BRCA1 or other DDR genes will be extremely RT sensitive.

### xCT inhibition provides tumor-specific sensitization

Our approach has several key advantages over previous sensitizer development work. First, it makes no assumption that glutathione is the only or dominant thiol which opposes radiation damage, although glutathione potently reduces death in our studies (Figure 7D, 7E). In fact recent studies demonstrate that inhibition of glutathione synthesis (via GGS inhibition), and inhibition of thioredoxin reductase produces more potent RT sensitization than inhibition of either enzyme alone [79]. Since all cellular thiols rely on cysteine for biosynthesis or activity, these observations reinforce our hypothesis that limiting cysteine availability will be a superior sensitization strategy, in agreement with historical literature demonstrating that cysteine was a superior, externally-applied radiation protectant [19]. Secondly, the limited normal tissue distribution of xCT, unlike GGS, thioredoxin reductase, and other potential sensitizing targets, suggests that xCT-mediated sensitization will be highly tumor-specific. Normal xCT^−^ tissues, both quiescent and proliferating will be spared, producing far fewer side effects than other proposed sensitizers. While clinically-approved xCT inhibitors are not yet available, our data speaks to the need for rapid development of such drugs.

## MATERIALS AND METHODS

### Cell culture and assays

Human breast cancer cell lines MDA-MB-436 (M436), MDA-MB-231 (M231), and MCF7 were obtained from Dr. Joe Gray (Oregon Health Sciences University), and maintained at 37°C / 5% CO_2_ in 5% Fetal Bovine Serum (FBS) supplemented DMEM or RPMI. Cultures were treated with vehicle (DMSO) or erastin as indicated. *SLC7A11* deleted lines were maintained and experiments established with 50 μM 2-mercaptoethanol (2-me) media addition, to allow uptake of cystine via transporters other than xCT. Glutathione was used at 5mM. Cells were seeded into 100mm dishes or 6 well plates and allowed to attach for 24 hours prior to treatment. Subsequently, cells were treated with DMSO/erastin, or washed (PBS) and cultured in cystine free media, or media without 2-me for 16 hours, followed by γ-radiation or sham treatment.

### Colony survival assay

After 10-20 days of incubation, colonies were 4% formalin fixed, stained (0.4 % crystal violet), and colonies with > 50 cells counted. Plating efficiency was calculated: # colonies counted / # cells plated. Surviving fraction = colonies counted/ (cells seeded x plating efficiency / 100). Using this information, response curves were generated and analyzed using the linear-quadratic formula “Surviving fraction = exp (αDose+βDose^2^)”; [80]. The dose that kills all but 10% of cells in a population is derived from this equation by setting the surviving fraction to 10% (0.1). The dose enhancement ratio for 10% survival (DER_10_), the statistic commonly reported in radiation sensitization studies, is the ratio of the calculated radiation dose for DMSO pre-treatment / radiation dose for erastin pretreatment. DER_10_>1 indicates that the treatment sensitizes cells.

### γ-Irradiation

All experiments used a J L Shepherd Mark I model-20 ^137^Cesium source irradiator.

### RNA extraction and RT-qPCR

Total RNA was isolated (RNA mini kit, QIAGEN) and reverse transcribed (iScript, Biorad) following manufacturer's directions. cDNA levels of *SLC7A11* were quantified in triplicate using Power SYBR green (AB). Data collection was performed on the Step One Plus (AB) sequence detection system. Data was quantified against a standard curve and normalized to TBP (TATA box binding protein) expression. Primers used were: *SLC7A11*: 5′ TGCTGGGCTGATTTTATCTTCG, 5′ GAAAGGGCAACCATGAAGAGG; TBP: 5′ CCC GAAACGCCGAATATAATCC, 5′ GACTGTTCTTCACT CTTGGCTC.

### Western blot

RIPA extracts were prepared by standard techniques in the presence of protease and phosphatase inhibitors (Sigma). 20 μg of lysates were resolved on 10% SDS-polyacrylamide gels, blotted to PVDF membrane, blocked for 1 hour in 5% nonfat dry milk/TBST 1 hour and exposed overnight 4°C to primary antibodies (anti-xCT, Cell Signaling and anti-β-actin, Sigma) following the manufacturer's instructions. After incubation with anti-rabbit-HRP conjugated antibodies, membranes were developed using ECL (Pierce 32209).

### Glutamate secretion

Erastin at indicated concentrations was added to fresh media without phenol red. The supernatants were collected 24 hours later and analyzed using Amplex Red Glutamic Acid/Glutamate Oxidase Assay Kit (Life Technologies) following manufacturer's instructions. Values were subtracted from media controls and normalized to cell number.

### Intracellular glutathione

Cells were plated in 96-well plates and treated with erastin for 24 hours. Intracellular glutathione was determined using GSH-Glo kit (Promega) following manufacturer's instructions. Values were normalized to cell number.

### Animal experiments

8-10 week old female NSG (NOD.Cg-*Prkdc^scid^ Il2rg^tm1Wjl^*/SzJ) mice from the Preclinical Therapeutics CORE at UCSF were used, following protocols approved by the Institutional Animal Care and Use Committee at UCSF, # AN142193-02A. Orthotopic injections of 10^6^ M436 cells in 100ul saline were made into fatpad 4. Tumors were measured (caliper) and mice weighed 3 times/week throughout the experimental time course. Tumor volume was calculated V= ((length X width^2^)/2). When tumors reached ~250 mm^3^, 4 groups of 5 mice each with equal total tumor volume were formed, and treatment started. Erastin stock was prepared in DMSO to 100mM, re-suspended by incubation in a sonic water bath cleaner, and aliquots frozen −80C. Daily peri-tumoral injections used erastin stock diluted in saline/1% Tween-20, to deliver 16.5 mg/kg erastin in 100ul or saline/1% Tween-20 control. Irradiation: Once after 24 hour erastin or saline pretreatment, 4 Gy partial body irradiation in a custom shielding apparatus, or sham treatment. Tumors were weighed upon dissection before preservation. We observed no overt signs of distress or discomfort in the animals throughout the duration of these experiments, and measured little weight loss.

### FACS analyses

A BD FACSCalibur or Accuri C6 flow cytometer was used. Experiments were performed at least twice, with 3-6 replicates per condition, and 10,000- 50,000 cells collected per sample, analyzed via the FLOJO software package. Cell cycle: Cells were fixed with cold 70% ethanol overnight, washed in PBS/5% FBS, stained in PBS/5% FBS with 10μg/ml Propidium Iodide (PI; Molecular Probes), and 100 μg/ml of RNase A. Annexin V staining: cells were stained with Annexin V- FITC (Southern Biotechnology 10038-02) in Annexin staining buffer. PI (Molecular Probes, P1304MP) was added just before analysis per manufacturer's instructions. ROS Detection: cultures were incubated for 15-30 min with 10 μM 2′,7′-dichlorofluorescein diacetate (DCFH-DA, Sigma) washed (PBS), harvested (trypsin), and analyzed.

### *SLC7A11* knockout generation

The *SLC7A11* gene was targeted using CRISPR/Cas9 technology. M436 and M231 cell lines were transfected with *SLC7A11* Double Nickase Plasmid (h) (Santa Cruz Biotechnology) using Lipofectamine (Invitrogen). Clones with specific mutations in the *SLC7A11* locus were puromicyn selected (0.5 μg/ml for 3 days), sorted for GFP expression and plated clonally. *SLC7A11* depletion was validated by western blot. Individual clones were maintained separately, and pooled for experimental analyses.

### Immunofluorescence

Cells grown and treated on glass coverslips were PBS washed, fixed (4% paraformaldehyde; PFA), permeabilized with PBS /0.1% Triton X-100/10 min., and blocked in PBS /1% bovine serum albumin (BSA)/ 5% goat serum. Primary antibodies: anti-γ-H2AX (Ser139; 20E3) and anti-53BP1 (Cell Signaling Technology) were incubated overnight at 4°C in block. Secondary: anti-rabbit Alexa 488 (Molecular Probes) / 1 hour with 4′,6-diamidino-2-phenylindole, dihydrochloride (DAPI) nuclear counterstain. At least 50 nuclei per condition were evaluated. For nuclear morphology assessment at day 5, cells were stained with DAPI (nuclei), and anti-CD44 (Thermo Scientific MA1-10225; cell cytoplasm and boundaries). 1000 nuclei per condition were evaluated.

### Statistical analysis

Glutamate secretion, GSH assay, ROS detection, nuclear foci counts, cell cycle analysis and Annexin V staining are presented as mean ± standard deviation, with significance via Student *t* test. For clonogenic survival, a linear regression model was used as previously described [81], and described above. Significant differences in the frequency of micronuclei and multiple nuclei, were determined by X^2^ test. Statistical analysis used SPSS 15.0 (SPSS Inc.), statistical significance was established when p ≤ 0.05. Boxplots in Figure 3F were generated using the web based version of BoxPlotR (http://shiny.chemgrid.org/boxplotr/).

## SUPPLEMENTARY MATERIALS FIGURES



## Abbreviations

RT: Radiation Therapy
ROS: Reactive Oxygen Species
IR: Ionizing Radiation
DDR: DNA Damage Response
DSB: Double Strand Breaks
NHEJ: Non-Homologous End Joining
HR: Homologous Recombination
BRCA1: Breast Cancer Associated 1
GSH: Reduced Glutathione
GSSG: Oxidized Glutathione
SASP: Sulfasalazine
TNBC: Triple Negative Breast Cancer
2-me: Beta-mercaptoethanol
DMSO: Dimethyl Sulfoxide
PBS: Phosphate Buffered Saline
DER_10_: Radiation Dose Required to Kill All But 10% of Cells in a Culture
RIPA: Radioimmunoprecipitation assay buffer
SDS: Sodium Dodecyl Sulfate
PVDF: Polyvinylidene Difluoride
HRP: Horseradish Peroxidase
ECL: Enhanced Chemiluminescence
FBS: Fetal Bovine Serum
PI: Propidium Iodide
DCFH-DA: 2′, 7′-Dichlorodihydrofluorescein Diacetate
GFP: Green Fluorescent Protein
PFA: Paraformaldehyde
DAPI: 4′,6-diamidino-2-phenylindole
SSB: Single Strand Break

## References

[1] Bryant AK, Banegas MP, Martinez ME, Mell LK, Murphy JD (2017). Trends in radiation therapy among cancer survivors in the united states, 2000-2030. Cancer Epidemiol Biomarkers Prev.

[2] Siegel R, DeSantis C, Virgo K, Stein K, Mariotto A, Smith T, Cooper D, Gansler T, Lerro C, Fedewa S, Lin C, Leach C, Cannady RS (2012). Cancer treatment and survivorship statistics, 2012. CA Cancer J Clin.

[3] Borras JM, Lievens Y, Barton M, Corral J, Ferlay J, Bray F, Grau C (2016). How many new cancer patients in Europe will require radiotherapy by 2025? An ESTRO-HERO analysis. Radiother Oncol.

[4] Begg AC, Stewart FA, Vens C (2011). Strategies to improve radiotherapy with targeted drugs. Nat Rev Cancer.

[5] Berkey FJ (2010). Managing the adverse effects of radiation therapy. Am Fam Physician.

[6] Yi A, Kim HH, Shin HJ, Huh MO, Ahn SD, Seo BK (2009). Radiation-induced complications after breast cancer radiation therapy: a pictorial review of multimodality imaging findings. Korean J Radiol.

[7] Tailby E, Boyages Am J (2017). Conservation surgery and radiation therapy in early breast cancer - An update. Aust Fam Physician.

[8] Yahyapour R, Motevaseli E, Rezaeyan A, Abdollahi H, Farhood B, Cheki M, Rezapoor S, Shabeeb D, Musa AE, Najafi M, Villa V (2018). Reduction-oxidation (redox) system in radiation-induced normal tissue injury: molecular mechanisms and implications in radiation therapeutics. Clin Transl Oncol.

[9] Ward JF (1988). DNA damage produced by ionizing radiation in mammalian cells: identities, mechanisms of formation, and reparability. Prog Nucleic Acid Res Mol Biol.

[10] Ciccia A, Elledge SJ (2010). The DNA damage response: making it safe to play with knives. Mol Cell.

[11] Bunting SF, Nussenzweig A (2013). End-joining, translocations and cancer. Nat Rev Cancer.

[12] Vitale I, Galluzzi L, Castedo M, Kroemer G (2011). Mitotic catastrophe: a mechanism for avoiding genomic instability. Nat Rev Mol Cell Biol.

[13] Kreuzaler P, Watson CJ (2012). Killing a cancer: what are the alternatives?. Nat Rev Cancer.

[14] Rogakou EP, Pilch DR, Orr AH, Ivanova VS, Bonner WM (1998). DNA double-stranded breaks induce histone H2AX phosphorylation on serine 139. J Biol Chem.

[15] Panier S, Boulton SJ (2014). Double-strand break repair: 53BP1 comes into focus. Nat Rev Mol Cell Biol.

[16] Bonner WM, Redon CE, Dickey JS, Nakamura AJ, Sedelnikova OA, Solier S, Pommier Y (2008). GammaH2AX and cancer. Nat Rev Cancer.

[17] Jones DP (2008). Radical-free biology of oxidative stress. Am J Physiol Cell Physiol.

[18] Schafer FQ, Buettner GR (2001). Redox environment of the cell as viewed through the redox state of the glutathione disulfide/glutathione couple. Free Radic Biol Med.

[19] Patt HM, Tyree EB, Straube RL, Smith DE (1949). Cysteine protection against X irradiation. Science.

[20] Brown DQ, Yuhas JM, MacKenzie LJ, Graham WJ, Pittock JW (1984). Differential radioprotection of normal tissues by hydrophilic chemical protectors. Int J Radiat Oncol Biol Phys.

[21] Bannai S, Tateishi N (1986). Role of membrane transport in metabolism and function of glutathione in mammals. J Membr Biol.

[22] Bannai S (1984). Transport of cystine and cysteine in mammalian cells. Biochim Biophys Acta.

[23] Habib E, Linher-Melville K, Lin HX, Singh G (2015). Expression of xCT and activity of system xc(−) are regulated by NRF2 in human breast cancer cells in response to oxidative stress. Redox Biol.

[24] Nioi P, Nguyen T (2007). A mutation of Keap1 found in breast cancer impairs its ability to repress Nrf2 activity. Biochem Biophys Res Commun.

[25] Shibata T, Ohta T, Tong KI, Kokubu A, Odogawa R, Tsuta K, Asamura H, Yamamoto M, Hirohashi S (2008). Cancer related mutations in NRF2 impair its recognition by Keap1-Cul3 E3 ligase and promote malignancy. Proc Natl Acad Sci U S A.

[26] Singh A, Misra V, Thimmulappa RK, Lee H, Ames S, Hoque MO, Herman JG, Baylin SB, Sidransky D, Gabrielson E, Brock MV, Biswal S (2006). Dysfunctional KEAP1-NRF2 interaction in non-small-cell lung cancer. PLoS Med.

[27] Shibata T, Kokubu A, Saito S, Narisawa-Saito M, Sasaki H, Aoyagi K, Yoshimatsu Y, Tachimori Y, Kushima R, Kiyono T, Yamamoto M (2011). NRF2 mutation confers malignant potential and resistance to chemoradiation therapy in advanced esophageal squamous cancer. Neoplasia.

[28] Shibata T, Kokubu A, Gotoh M, Ojima H, Ohta T, Yamamoto M, Hirohashi S (2008). Genetic alteration of Keap1 confers constitutive Nrf2 activation and resistance to chemotherapy in gallbladder cancer. Gastroenterology.

[29] Singh A, Bodas M, Wakabayashi N, Bunz F, Biswal S (2010). Gain of Nrf2 function in non-small-cell lung cancer cells confers radioresistance. Antioxid Redox Signal.

[30] Yang Y, Yee D (2014). IGF-I regulates redox status in breast cancer cells by activating the amino acid transport molecule xC. Cancer Res.

[31] Lewerenz J, Maher P (2009). Basal levels of eIF2alpha phosphorylation determine cellular antioxidant status by regulating ATF4 and xCT expression. J Biol Chem.

[32] Sato H, Nomura S, Maebara K, Sato K, Tamba M, Bannai S (2004). Transcriptional control of cystine/glutamate transporter gene by amino acid deprivation. Biochem Biophys Res Commun.

[33] Uhlen M, Fagerberg L, Hallstrom BM, Lindskog C, Oksvold P, Mardinoglu A, Sivertsson A, Kampf C, Sjostedt E, Asplund A, Olsson I, Edlund K, Lundberg E (2015). Proteomics. Tissue-based map of the human proteome. Science.

[34] The Human Protein Atlas, SLC7A11 gene specific mRNA data and figures in normal tissues. https://www.proteinatlas.org/ENSG00000151012-SLC7A11/tissue.

[35] Chintala S, Li W, Lamoreux ML, Ito S, Wakamatsu K, Sviderskaya EV, Bennett DC, Park YM, Gahl WA, Huizing M, Spritz RA, Ben S, Novak EK (2005). Slc7a11 gene controls production of pheomelanin pigment and proliferation of cultured cells. Proc Natl Acad Sci U S A.

[36] Sato H, Shiiya A, Kimata M, Maebara K, Tamba M, Sakakura Y, Makino N, Sugiyama F, Yagami K, Moriguchi T, Takahashi S, Bannai S (2005). Redox imbalance in cystine/glutamate transporter-deficient mice. J Biol Chem.

[37] Nabeyama A, Kurita A, Asano K, Miyake Y, Yasuda T, Miura I, Nishitai G, Arakawa S, Shimizu S, Wakana S, Yoshida H, Tanaka M (2010). xCT deficiency accelerates chemically induced tumorigenesis. Proc Natl Acad Sci U S A.

[38] Robert SM, Buckingham SC, Campbell SL, Robel S, Holt KT, Ogunrinu-Babarinde T, Warren PP, White DM, Reid MA, Eschbacher JM, Berens ME, Lahti AC, Nabors LB (2015). SLC7A11 expression is associated with seizures and predicts poor survival in patients with malignant glioma. Sci Transl Med.

[39] Zhang RR, Pointer KB, Kuo JS (2015). Excitotoxic SLC7A11 Expression Is a Marker of Poor Glioblastoma survival and a potential therapeutic target. Neurosurgery.

[40] Takeuchi S, Wada K, Toyooka T, Shinomiya N, Shimazaki H, Nakanishi K, Nagatani K, Otani N, Osada H, Uozumi Y, Matsuo H, Nawashiro H (2013). Increased xCT expression correlates with tumor invasion and outcome in patients with glioblastomas. Neurosurgery.

[41] Shiozaki A, Iitaka D, Ichikawa D, Nakashima S, Fujiwara H, Okamoto K, Kubota T, Komatsu S, Kosuga T, Takeshita H, Shimizu H, Nako Y, Sasagawa H (2014). xCT, component of cysteine/glutamate transporter, as an independent prognostic factor in human esophageal squamous cell carcinoma. J Gastroenterol.

[42] Kinoshita H, Okabe H, Beppu T, Chikamoto A, Hayashi H, Imai K, Mima K, Nakagawa S, Ishimoto T, Miyake K, Yokoyama N, Ishiko T, Baba H (2013). Cystine/glutamic acid transporter is a novel marker for predicting poor survival in patients with hepatocellular carcinoma. Oncol Rep.

[43] Namikawa M, Kakizaki S, Kaira K, Tojima H, Yamazaki Y, Horiguchi N, Sato K, Oriuchi N, Tominaga H, Sunose Y, Nagamori S, Kanai Y, Oyama T (2015). Expression of amino acid transporters (LAT1, ASCT2 and xCT) as clinical significance in hepatocellular carcinoma. Hepatol Res.

[44] Sugano K, Maeda K, Ohtani H, Nagahara H, Shibutani M, Hirakawa K (2015). Expression of xCT as a predictor of disease recurrence in patients with colorectal cancer. Anticancer Res.

[45] Wang X, Huang P, Yang S, Wahl R (2015). Receiver operating characteristic (ROC) analysis of amino acid transporters in 136 prostate cancer samples. Journal of Nuclear Medicine.

[46] Ji XJ, Qian J, Rahman J, Harris B, Hoeksema M, Chen H, Eisenberg R, Young J (2016). Abstract A10: SLC7A11 contributes to the pathogenesis of lung cancer. Molecular Cancer Research.

[47] Timmerman LA, Holton T, Yuneva M, Louie RJ, Padro M, Daemen A, Hu M, Chan DA, Ethier SP, van 't Veer LJ, Polyak K, McCormick F, Gray JW (2013). Glutamine sensitivity analysis identifies the xCT antiporter as a common triple-negative breast tumor therapeutic target. Cancer Cell.

[48] Forbes SA, Beare D, Boutselakis H, Bamford S, Bindal N, Tate J, Cole CG, Ward S, Dawson E, Ponting L, Stefancsik R, Harsha B, Kok CY (2017). COSMIC: somatic cancer genetics at high-resolution. Nucleic Acids Res.

[49] Elstrodt F, Hollestelle A, Nagel JH, Gorin M, Wasielewski M, van den Ouweland A, Merajver SD, Ethier SP, Schutte M (2006). BRCA1 mutation analysis of 41 human breast cancer cell lines reveals three new deleterious mutants. Cancer Res.

[50] Ishii T, Bannai S, Sugita Y (1981). Mechanism of growth stimulation of L1210 cells by 2-mercaptoethanol *in vitro*. Role of the mixed disulfide of 2-mercaptoethanol and cysteine. J Biol Chem.

[51] Dixon SJ, Lemberg KM, Lamprecht MR, Skouta R, Zaitsev EM, Gleason CE, Patel DN, Bauer AJ, Cantley AM, Yang WS, Morrison B, Stockwell BR (2012). Ferroptosis: an iron-dependent form of nonapoptotic cell death. Cell.

[52] Dixon SJ, Patel DN, Welsch M, Skouta R, Lee ED, Hayano M, Thomas AG, Gleason CE, Tatonetti NP, Slusher BS, Stockwell BR (2014). Pharmacological inhibition of cystine-glutamate exchange induces endoplasmic reticulum stress and ferroptosis. Elife.

[53] Vos O, Roos-Verhey WS (1984). Protection against X-irradiation by some orally administered compounds. Int J Radiat Biol Relat Stud Phys Chem Med.

[54] Vos O, Roos-Verhey WS (1988). Radioprotection by glutathione esters and cysteamine in normal and glutathione-depleted mammalian cells. Int J Radiat Biol Relat Stud Phys Chem Med.

[55] Citrin D, Cotrim AP, Hyodo F, Baum BJ, Krishna MC, Mitchell JB (2010). Radioprotectors and mitigators of radiation-induced normal tissue injury. Oncologist.

[56] Kim K, Pollard JM, Norris AJ, McDonald JT, Sun Y, Micewicz E, Pettijohn K, Damoiseaux R, Iwamoto KS, Sayre JW, Price BD, Gatti RA, McBride WH (2009). High-throughput screening identifies two classes of antibiotics as radioprotectors: tetracyclines and fluoroquinolones. Clin Cancer Res.

[57] Camphausen K, Citrin D, Krishna MC, Mitchell JB (2005). Implications for tumor control during protection of normal tissues with antioxidants. J Clin Oncol.

[58] Meister A, Tate SS (1976). Glutathione and related gamma-glutamyl compounds: biosynthesis and utilization. Annu Rev Biochem.

[59] Seiwert TY, Salama JK, Vokes EE (2007). The concurrent chemoradiation paradigm—general principles. Nat Clin Pract Oncol.

[60] Wardman P (2007). Chemical radiosensitizers for use in radiotherapy. Clin Oncol (R Coll Radiol).

[61] Du S, Bouquet S, Lo CH, Pellicciotta I, Bolourchi S, Parry R, Barcellos-Hoff MH (2015). Attenuation of the DNA damage response by transforming growth factor-beta inhibitors enhances radiation sensitivity of non-small-cell lung cancer cells *in vitro* and *in vivo*. Int J Radiat Oncol Biol Phys.

[62] Park CC, Zhang HJ, Yao ES, Park CJ, Bissell MJ (2008). Beta1 integrin inhibition dramatically enhances radiotherapy efficacy in human breast cancer xenografts. Cancer Res.

[63] Horsman MR, Overgaard J (2016). The impact of hypoxia and its modification of the outcome of radiotherapy. J Radiat Res.

[64] Bump EA, Yu NY, Brown JM (1982). Radiosensitization of hypoxic tumor cells by depletion of intracellular glutathione. Science.

[65] Lewerenz J, Hewett SJ, Huang Y, Lambros M, Gout PW, Kalivas PW, Massie A, Smolders I, Methner A, Pergande M, Smith SB, Ganapathy V, Maher P (2013). The cystine/glutamate antiporter system x(c)(−) in health and disease: from molecular mechanisms to novel therapeutic opportunities. Antioxid Redox Signal.

[66] Yagoda N, von Rechenberg M, Zaganjor E, Bauer AJ, Yang WS, Fridman DJ, Wolpaw AJ, Smukste I, Peltier JM, Boniface JJ, Smith R, Lessnick SL, Sahasrabudhe S (2007). RAS-RAF-MEK-dependent oxidative cell death involving voltage-dependent anion channels. Nature.

[67] Sleire L, Skeie BS, Netland IA, Forde HE, Dodoo E, Selheim F, Leiss L, Heggdal JI, Pedersen PH, Wang J, Enger PO (2015). Drug repurposing: sulfasalazine sensitizes gliomas to gamma knife radiosurgery by blocking cystine uptake through system Xc-, leading to glutathione depletion. Oncogene.

[68] Habens F, Srinivasan N, Oakley F, Mann DA, Ganesan A, Packham G (2005). Novel sulfasalazine analogues with enhanced NF-kB inhibitory and apoptosis promoting activity. Apoptosis.

[69] Weber CK, Liptay S, Wirth T, Adler G, Schmid RM (2000). Suppression of NF-kappaB activity by sulfasalazine is mediated by direct inhibition of IkappaB kinases alpha and beta. Gastroenterology.

[70] Salerno KE (2017). NCCN guidelines update: evolving radiation therapy recommendations for breast cancer. J Natl Compr Canc Netw.

[71] Ward IM, Chen J (2001). Histone H2AX is phosphorylated in an ATR-dependent manner in response to replicational stress. J Biol Chem.

[72] de Feraudy S, Revet I, Bezrookove V, Feeney L, Cleaver JE (2010). A minority of foci or pan-nuclear apoptotic staining of gammaH2AX in the S phase after UV damage contain DNA double-strand breaks. Proc Natl Acad Sci U S A.

[73] Rydberg B (1996). Clusters of DNA damage induced by ionizing radiation: formation of short DNA fragments. II. Experimental detection. Radiat Res.

[74] Goodhead DT (1994). Initial events in the cellular effects of ionizing radiations: clustered damage in DNA. Int J Radiat Biol.

[75] Groesser T, Chang H, Fontenay G, Chen J, Costes SV, Helen Barcellos-Hoff M, Parvin B, Rydberg B (2011). Persistence of gamma-H2AX and 53BP1 foci in proliferating and non-proliferating human mammary epithelial cells after exposure to gamma-rays or iron ions. Int J Radiat Biol.

[76] Klokov D, MacPhail SM, Banath JP, Byrne JP, Olive PL (2006). Phosphorylated histone H2AX in relation to cell survival in tumor cells and xenografts exposed to single and fractionated doses of X-rays. Radiother Oncol.

[77] Banath JP, Klokov D, MacPhail SH, Banuelos CA, Olive PL (2010). Residual gammaH2AX foci as an indication of lethal DNA lesions. BMC Cancer.

[78] Lobrich M, Cooper PK, Rydberg B (1996). Non-random distribution of DNA double-strand breaks induced by particle irradiation. Int J Radiat Biol.

[79] Rodman SN, Spence JM, Ronnfeldt TJ, Zhu Y, Solst SR, O'Neill RA, Allen BG, Guan X, Spitz DR, Fath MA (2016). Enhancement of radiation response in breast cancer stem cells by inhibition of thioredoxin- and glutathione-dependent metabolism. Radiat Res.

[80] Fowler JF (1989). The linear-quadratic formula and progress in fractionated radiotherapy. Br J Radiol.

[81] Franken NA, Rodermond HM, Stap J, Haveman J, van Bree C (2006). Clonogenic assay of cells *in vitro*. Nat Protoc.

