# Supplementary material for: xCT inhibition sensitizes tumors to γ-radiation via glutathione reduction
Source: Oncotarget. 2018 Aug 17;9(64):32280–97. doi: 10.18632/oncotarget.25794 (PMC6122354; doi:10.18632/oncotarget.25794)
Supplement: Supplementary file 1 [file oncotarget-09-32280-s001.pdf]

xCT inhibition sensitizes tumors to γ-radiation via glutathione reduction

SUPPLEMENTARY MATERIALS

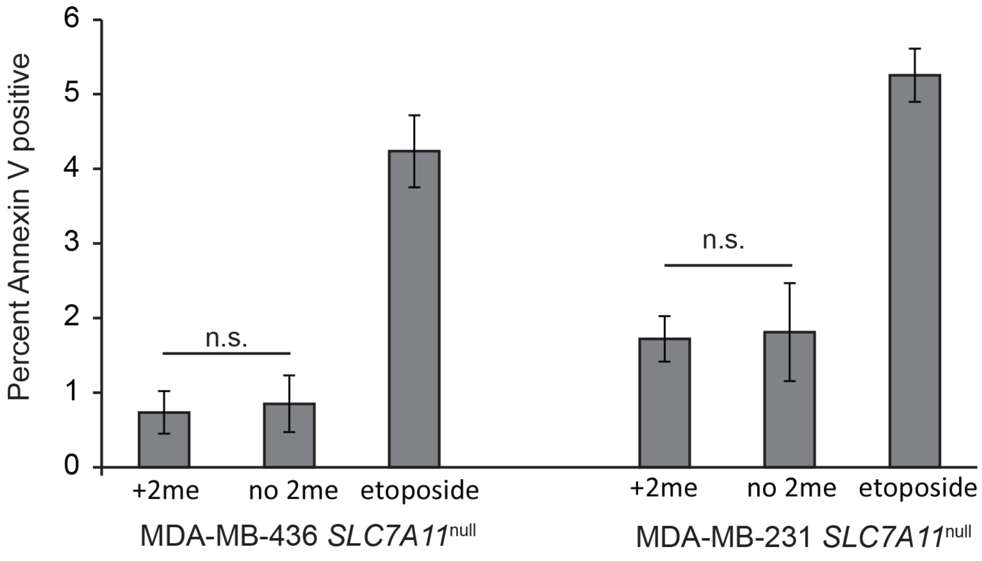

Supplementary Figure 1: *SLC7A11*null cells do not die when cultured for 24 hours without 2-me. Percent annexin V positive cells determined by annexin V /PI staining and FACS analysis of 10,000-50,000 cells per condition. Experiments performed 2-3 times with 6 replicates each. Etoposide treatment (100µM) used to provide an annexin V staining positive control. Values are averages of experimental means +/- SEM. *t*-test significance; n.s. not significant; *, p<=0.05; **, p<=0.01; ***, p<=0.001.

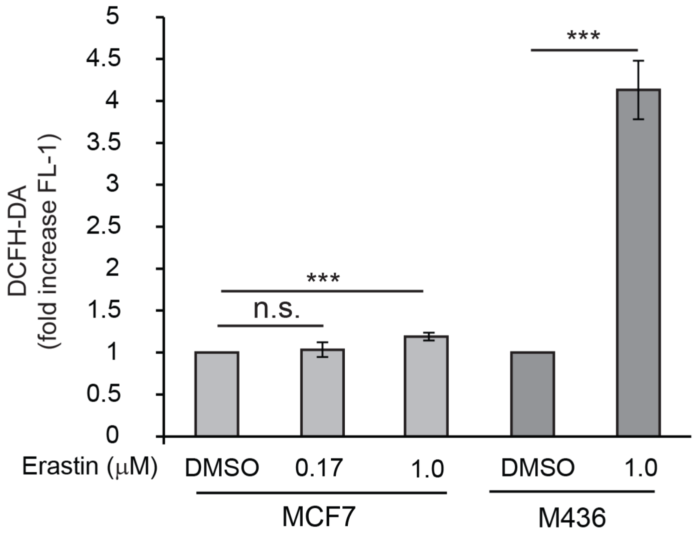

Supplementary Figure 2: The xCT negative cell line MCF7 induces little ROS increase upon erastin treatment. Cells were stained with the ROS detection reagent DCFH-DA (10µM) for 1 hour, washed and fluorescence measured (FL-1) by FACS. Experiments were done in at least triplicate with 20,000 cells analyzed per sample, and repeated 2-4 times. Representative experimental fold increase over DMSO treatment alone shown +/-S.D. Fold increase MCF7 with 0.17µM erastin = 1.0; fold increase with 1.0µM = 1.2; fold increase M436 with 1.0µM erastin = 4.1. *t*-test significance; n.s., not significant; *, p<=0.05; **, p<=0.01; ***, p<=0.001.

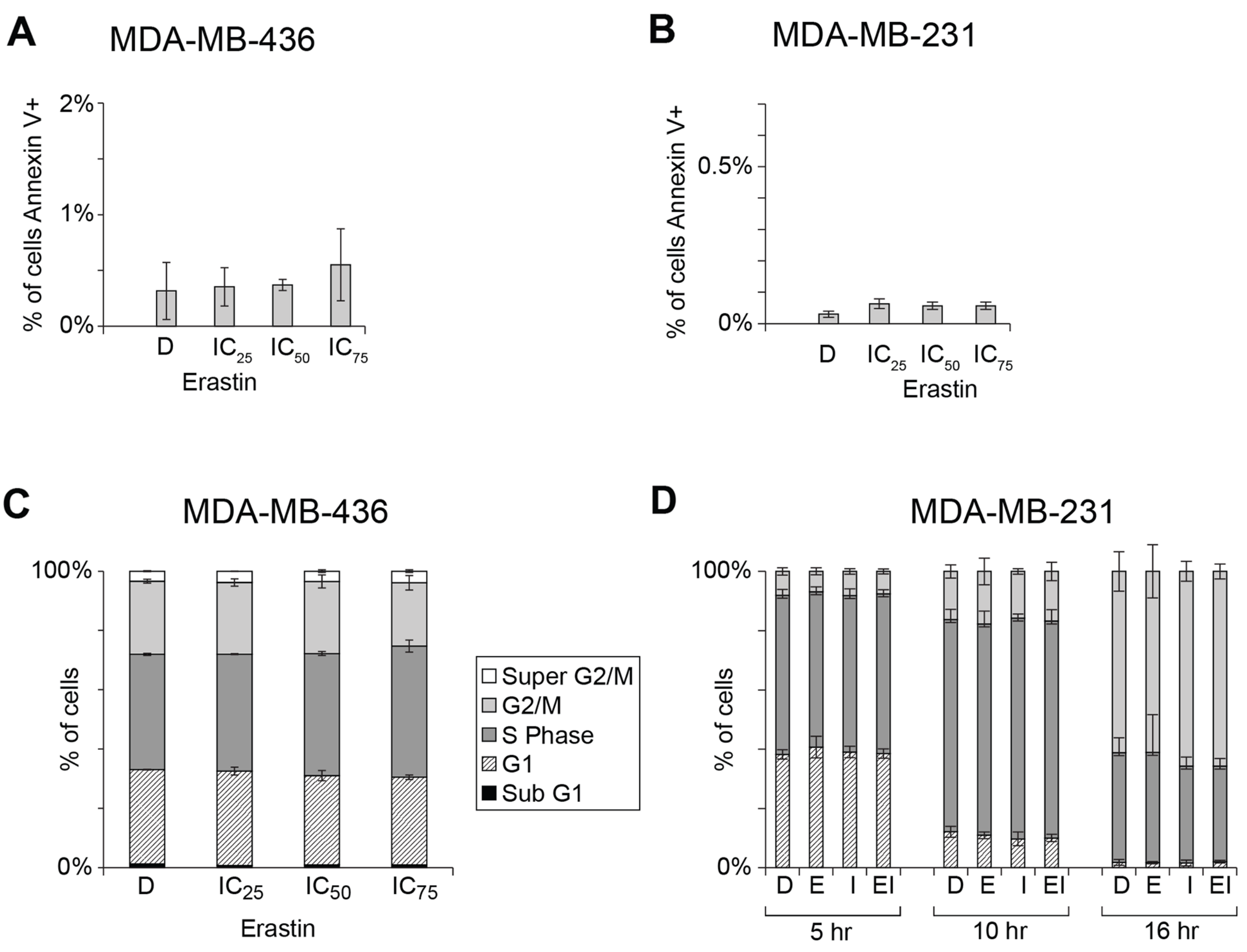

Supplementary Figure 3: Sixteen hours of erastin treatment does not increase cell death or alter cell cycle progression. **(A, B)**, Cell death measured by PI / Annexin V staining and FACS analysis. Erastin concentrations: MDA-MB-436 IC25, 0.66µM; IC50,1.0µM; IC75,1.66µM. MDA-MB-231, IC25,0.08µM, IC50, 0.17µM, IC75 0.33µM. D, DMSO control. **(C, D)** Cell cycle fractions determined by PI staining and data analysis using the FLOJO software package. D, DMSO control; E, erastin at IC50; I, 6 Gy. All experiments were done in at least triplicate with 50,000-100,000 cells analyzed per sample by FACS, and repeated 2-4 times, values presented are representative experimental means +/- SD.

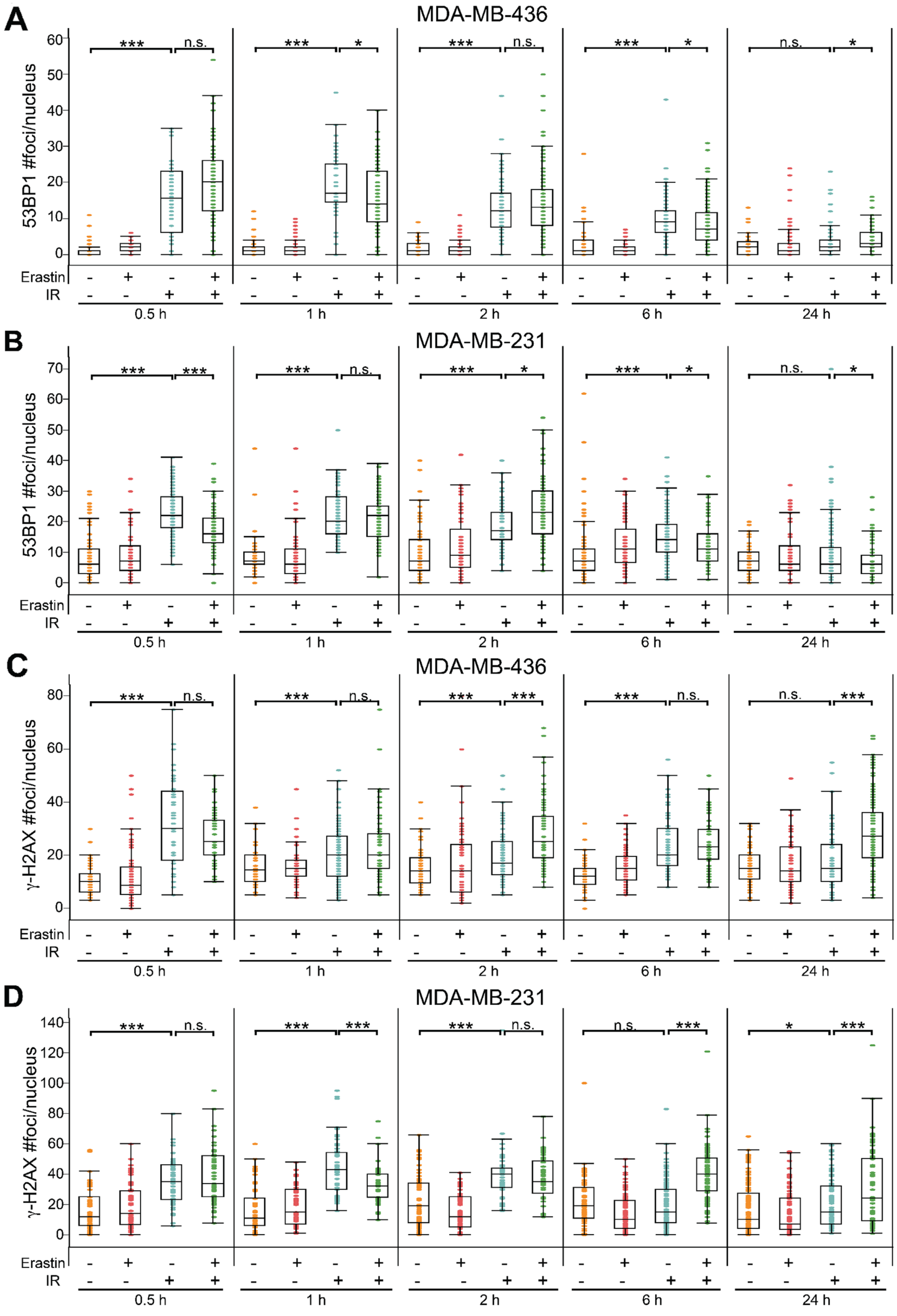

Supplementary Figure 4: Representative complete timecourse study of DNA repair foci numbers with and without erastin sensitization before IR treatment. MDA-MB-436 and MDA-MB-231 were pre-treated for 16 hours with erastin at the respective IC50s before 2 Gy IR. MDA-MB- 436 IC50,1.0μM; MDA-MB-231 IC50, 0.17μM. **(A, B)**, 53BP1 foci/nucleus. **(C, D)** γ H2AXfoci/nucleus. Foci assessed by immune fluorescence and microscopic quantitation. At least 50 nuclei per condition evaluated. Experiments performed in triplicate 2-3 times. Mann Whitney *U* test, significance; n.s. not significant; *, $p<=0.05$; **, $p<=0.01$; ***, $p<=0.001$.

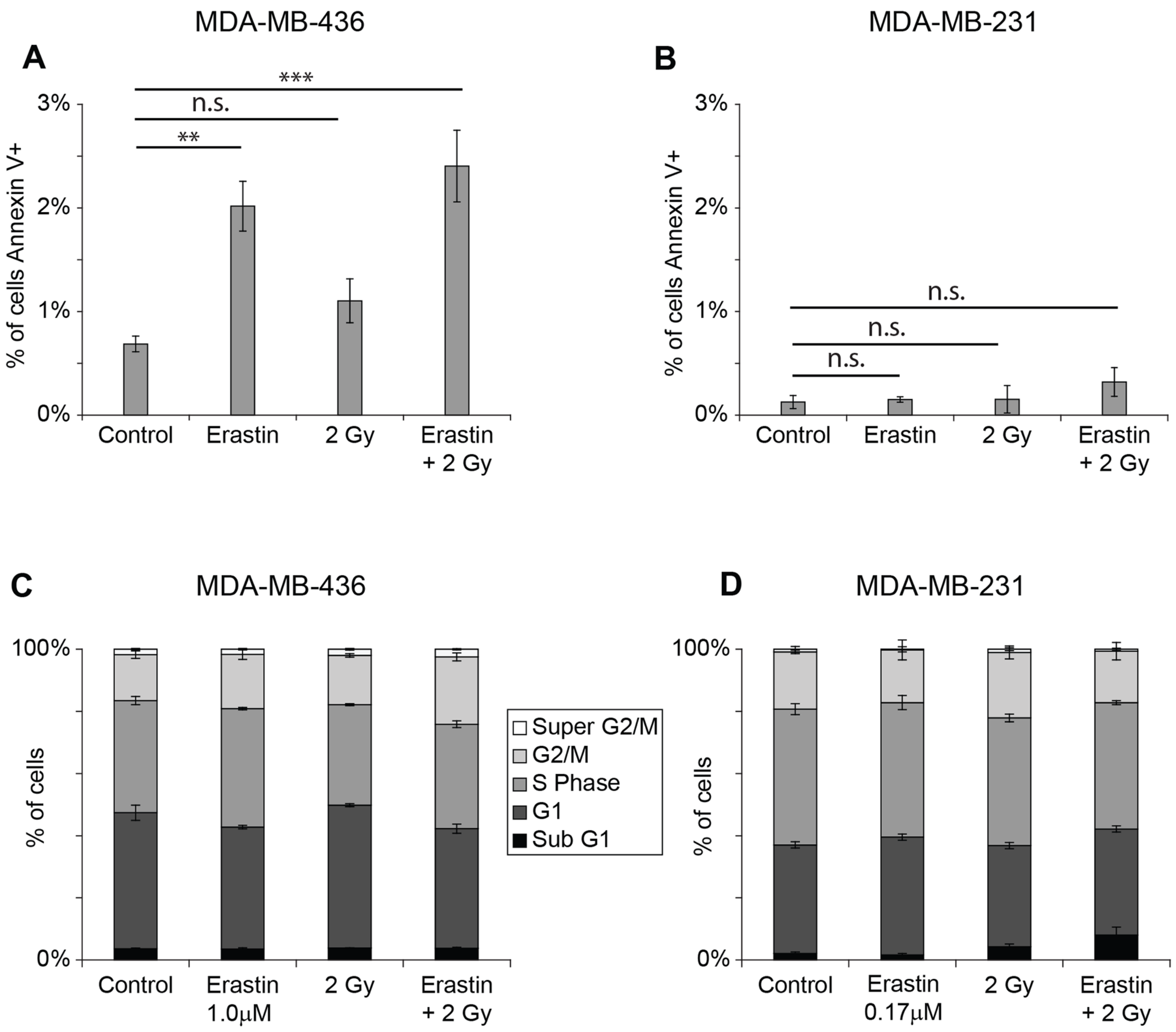

Supplementary Figure 5: Differences in γ-H2AX foci numbers 24 hours post irradiation are not attributable to differences in cell death or cell cycle distribution between treatment conditions. Cells were treated with erastin for 16 hours before 2 Gy IR. Erastin concentrations: MDA-MB-436, 1.0μM; MDA-MB-231, 0.17μM. **(A, B)** Cell death measured by PI / Annexin V staining and FACS analysis. **(C, D)** Cell cycle fractions determined by PI stain, FACS, and data analysis using the FLOJO software package. Experiments were done in at least triplicate 2-3 times with 20,000-100,000 cells analyzed per sample by FACS. Representative experimental means +/- SD shown. *t*-test significance; n.s., not significant; *, $p<=0.05$; **, $p<=0.01$; ***, $p<=0.001$.

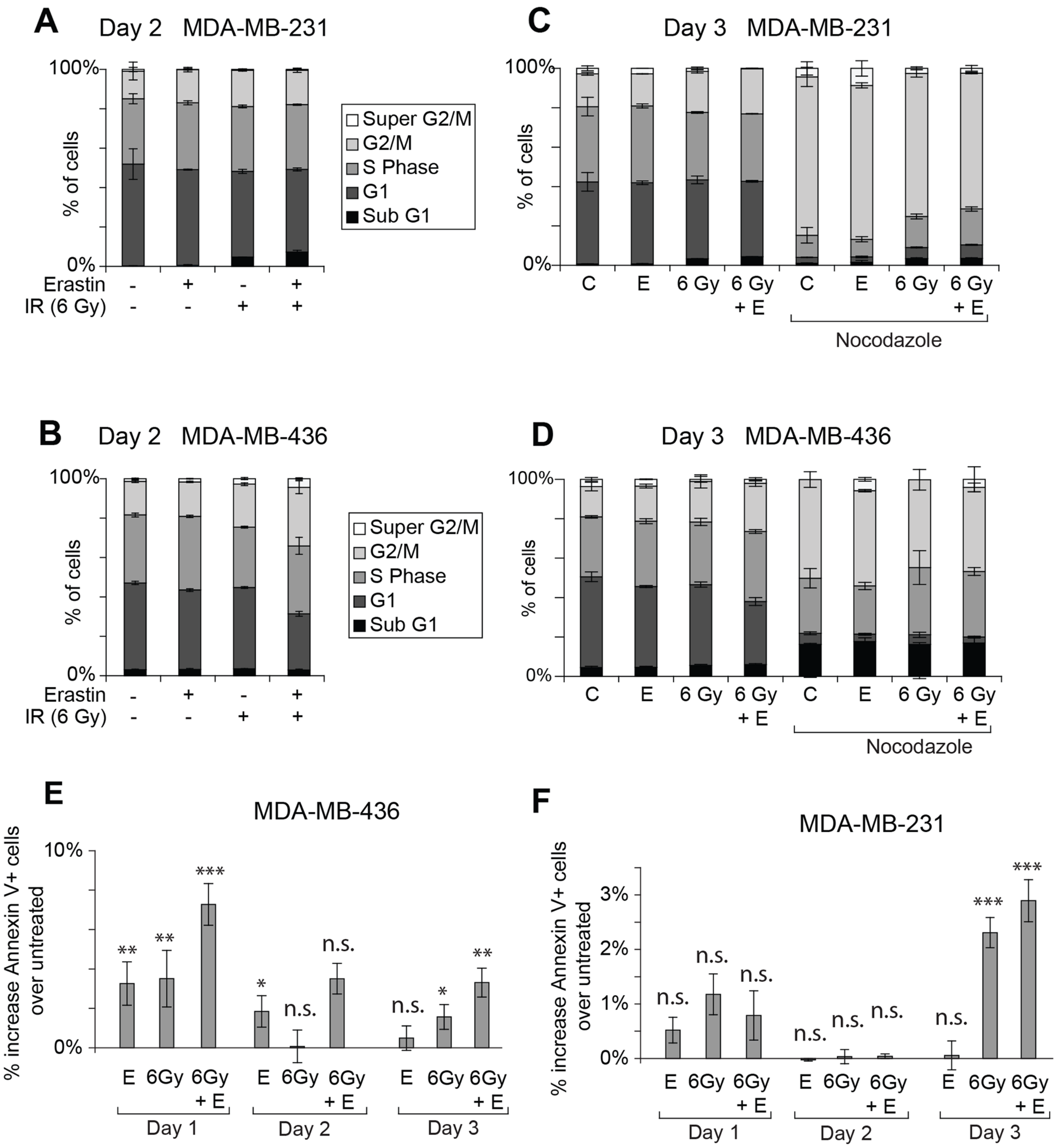

Supplementary Figure 6: Tumor cells are proliferating 3 days after irradiation, with little evidence of increased cell death. C, control cells (DMSO); E, Erastin at the IC 50 (MDA-MB-436, 1.0µM; MDA-MB-231, 0.17µM ; 6Gy, 6 Gray IR. All experiments performed in at least triplicate wtih 50,000-100,000 cells analyzed per sample, repeated 2-4 times. **(A, B)** Cell cycle profiles 2 days post IR. **(C, D)** expanded G2/M fractions from nocodazole treatment at day 3 reveals ongoing transit from G1 and S phases into G2M. Cell cycle fractions determined by PI staining, FACS, and FLOJO software package analysis. **(E, F)** Cells exhibit only minor increases in cell death 1-3 days post IR, with or without erastin sensitization. Cell death measured by PI/ annexin V staining and FACS analysis. All values are mean +/- SD. *t*-test significance; n.s., not significant; *, p<=0.05; ** p<= 0.01; ***, p<=0.001.
